# Tissue engineering potential of human dermis-isolated adult stem cells from multiple anatomical locations

**DOI:** 10.1371/journal.pone.0182531

**Published:** 2017-08-02

**Authors:** Heenam Kwon, Anne K. Haudenschild, Wendy E. Brown, Natalia Vapniarsky, Nikolaos K. Paschos, Boaz Arzi, Jerry C. Hu, Kyriacos A. Athanasiou

**Affiliations:** 1 Department of Biomedical Engineering, University of California, Davis, CA, United States of America; 2 Department of Orthopaedic Surgery, Penn Sports Medicine, University of Pennsylvania Health System, Philadelphia, PA, United States of America; 3 Department of Surgical and Radiological Sciences, School of Veterinary Medicine, University of California Davis, Davis, CA, United States of America; 4 Department of Orthopaedic Surgery, University of California Davis Medical Center, Sacramento, CA, United States of America; University of Pittsburgh, UNITED STATES

## Abstract

Abundance and accessibility render skin-derived stem cells an attractive cell source for tissue engineering applications. Toward assessing their utility, the variability of constructs engineered from human dermis-isolated adult stem (hDIAS) cells was examined with respect to different anatomical locations (foreskin, breast, and abdominal skin), both *in vitro* and in a subcutaneous, athymic mouse model. All anatomical locations yielded hDIAS cells with multi-lineage differentiation potentials, though adipogenesis was not seen for foreskin-derived hDIAS cells. Using engineered cartilage as a model, tissue engineered constructs from hDIAS cells were compared. Construct morphology differed by location. The mechanical properties of human foreskin- and abdominal skin-derived constructs were similar at implantation, remaining comparable after 4 additional weeks of culture *in vivo*. Breast skin-derived constructs were not mechanically testable. For all groups, no signs of abnormality were observed in the host. Addition of aggregate redifferentiation culture prior to construct formation improved chondrogenic differentiation of foreskin-derived hDIAS cells, as evident by increases in glycosaminoglycan and collagen contents. More robust Alcian blue staining and homogeneous cell populations were also observed compared to controls. Human DIAS cells elicited no adverse host responses, reacted positively to chondrogenic regimens, and possessed multi-lineage differentiation potential with the caveat that efficacy may differ by anatomical origin of the skin. Taken together, these results suggest that hDIAS cells hold promise as a potential cell source for a number of tissue engineering applications.

## Introduction

Human stem cells have gained interest from the tissue engineering community due to their multi-potency and their ample capacity for self-renewal [1]. Multiple research efforts have been dedicated to the selection of appropriate stem cell populations [2–4] and to the improvement of cell differentiation [5–7]. Pluripotent, embryonic stem cells face ethical concerns. Adult stem cells are often derived from neonates and these include cord-blood stem cells [8] and skin-derived stem cells of the foreskin [9]. Neither of these allow for autologous therapies for the existing, general population. Adult stem cells that hold potential for autologous therapies in tissue engineering include those isolated from human bone marrow [10], skin [11], adipose tissue [12], and blood [13]. Of these, cells that require minimally invasive isolation techniques are highly desired.

Human dermis-isolated adult stem (hDIAS) cells are attractive because they are easily accessible and are separate from the diseased or degenerated tissues that require repair. Small skin biopsies can potentially yield large cell numbers after expansion. Stem cells derived from the abdominal skin of the caprine model have shown promising results in generating articular cartilage [11]. Translating these technologies to humans would require, as a first step, examining whether differences exist for skin-derived stem cells as a function of anatomical location. Both abdominal and breast skins are easily accessible tissues, and therapies using these donor tissues would eliminate host rejection by allowing for autologous implants. While human foreskin-derived stem cells are limited to allogeneic therapies, they remain a viable potential cell source with promising results in tissue engineering [14]. Human stem cells of neonates have been shown to differentiate into cells of both the neuroectodermal and mesodermal lineages, and cell culture protocols have been developed to expand these progenitor cells without loss of multi-potency [14]. In short, comparison of skin-derived stem cells from various anatomical locations is a necessary first step toward translation.

Given the multiple types of tissues that stem cells can form, separate examinations should be performed for each tissue engineering application with medical need as a strong motivating factor. There is currently a strong demand for tissue-engineered cartilage. Articular cartilage degeneration resulting from post-traumatic injury or age-related degeneration is a major health problem worldwide [15]. Cartilage defects are incapable of undergoing self-repair and typically progress to osteoarthritis [16]. Currently, in the U.S. alone, over 46 million adults (21% of the population) suffer from osteoarthritis, resulting in an economic cost of over $80 billion a year [17]. These numbers are projected to increase steadily as the aged population grows to reach nearly 67 million sufferers by 2030 [18]. This enormous burden of disease has created a great clinical need for cartilage replacement and has motivated the development of various cartilage tissue engineering technologies over the past two decades. Combining these factors with existing knowledge of robust protocols for engineering articular cartilage [19] using differentiated chondrocytes of various species, this study uses articular cartilage as the model system to evaluate hDIAS cell utility.

Previous studies have been successful in fabricating functional engineered cartilages utilizing cells isolated from cartilage of various animal models in a scaffold-free, self-assembling process that recapitulates cartilage development [19, 20]. For example, primary articular chondrocytes isolated from juvenile bovine [21], ovine [22], and leporine [23] cartilage formed hyaline-like cartilage with biochemical and biomechanical values within the range of native articular cartilage. While the equilibrium compressive mechanical properties and glycosaminoglycan (GAG) content of constructs engineered using animal chondrocytes have reached values similar to native tissue [24], transitioning these promising results to the clinic requires an adequate number of human cells capable of forming mechanically robust replacement tissue. The early strategies that employed isolated human autologous chondrocytes [25, 26] from mature tissues faced challenges due to problems associated with preexisting degeneration of the donor cartilage, limited harvesting sites, donor site morbidity, and the limited expansion capacity of fully differentiated adult cells [27]. The utilization of hDIAS cells as an alternative to autologous chondrocytes for cartilage tissue engineering would address these issues, thus motivating this research.

The overall objective of this study was to examine the utility of human, skin-derived stem cells for tissue engineering. To do so, four main objectives were examined: The first objective was to compare the multi-lineage differentiation potential of stem cells derived from foreskin, breast skin, and abdominal skin of two donors per skin type. It was hypothesized that cells from all anatomical locations would display multi-lineage differentiation potential. The second objective used chondrogenesis as a model to compare the effects of anatomical location on self-assembled hDIAS cell construct properties. It was also hypothesized that the quality of neocartilage constructs formed would depend upon anatomical location. The third objective evaluated the *in vivo* stability, integrity, and safety of hDIAS cell-derived constructs from multiple anatomical sites. It was hypothesized that the subcutaneous environment would be sufficient to maintain phenotypic stability and construct mechanical integrity, and hDIAS-derived construct implantation would be deemed safe and, therefore, elicit no adverse reactions in the host animals. The fourth objective was to determine the efficacy of an aggregate redifferentiation culture (ARC) which has previously been shown to be chondroinductive in both animal cells and human marrow-derived stem cells on hDIAS cells. It was hypothesized that ARC would significantly improve both the mechanical and biochemical properties of self-assembled hDIAS cell constructs by chondrogenically priming the cells prior to construct formation.

## Materials and methods

### Cell isolation

De-identified human foreskin, breast skin, and abdominal skin discarded from procedures unrelated to this study were obtained from Cooperative Human Tissue Network (CHTN) Western Division (Vanderbilt University, Nashville, Tennessee) under an exemption determined by the UC Davis Institutional Review Board. All experiments were performed using two donors per anatomical location. Age, sex, and ethnicity of each skin type and donor are provided in S1 Table. Skins were washed in a “base medium” composed of Dulbecco’s Modified Eagle Medium (DMEM) with high glucose/GlutaMAX™-I (Life Technologies, Grand Island, NY) and 1% penicillin/streptomycin/fungizone (P/S/F) (Lonza, Basel, Switzerland), and the sub-dermal fat layer was removed. To remove the epidermis from the dermal tissue, the epidermis layer was scored with a customized cutter into 5 mm squares and soaked in base medium containing 0.2% dispase II (Roche, Indianapolis, IN) overnight at 4°C to facilitate penetration of the enzyme. After removing the epidermis layer, dermal tissues were minced and digested in a 0.25% pronase (Sigma-Aldrich, St. Louis, MO) solution containing 3% fetal bovine serum (FBS) (Atlanta Biologicals, Lawrenceville, GA) for 1 hour, followed by digestion in a 0.2% collagenase type II (Worthington, Lakewood, NJ) solution containing 2% FBS for 16–18 hours at 37°C. Following the digestion, cells were collected, filtered through 70 μm cell strainers, and washed 2–3 times with base medium. The isolated dermal cells were counted and frozen in freezing medium consisting of 90% FBS and 10% dimethyl sulfoxide (Sigma-Aldrich). Cells were isolated from two donors per anatomical location for *in vivo* studies and an additional foreskin donor was isolated for multi-lineage and ARC experiments.

### Cell processing

**Fig 1:**
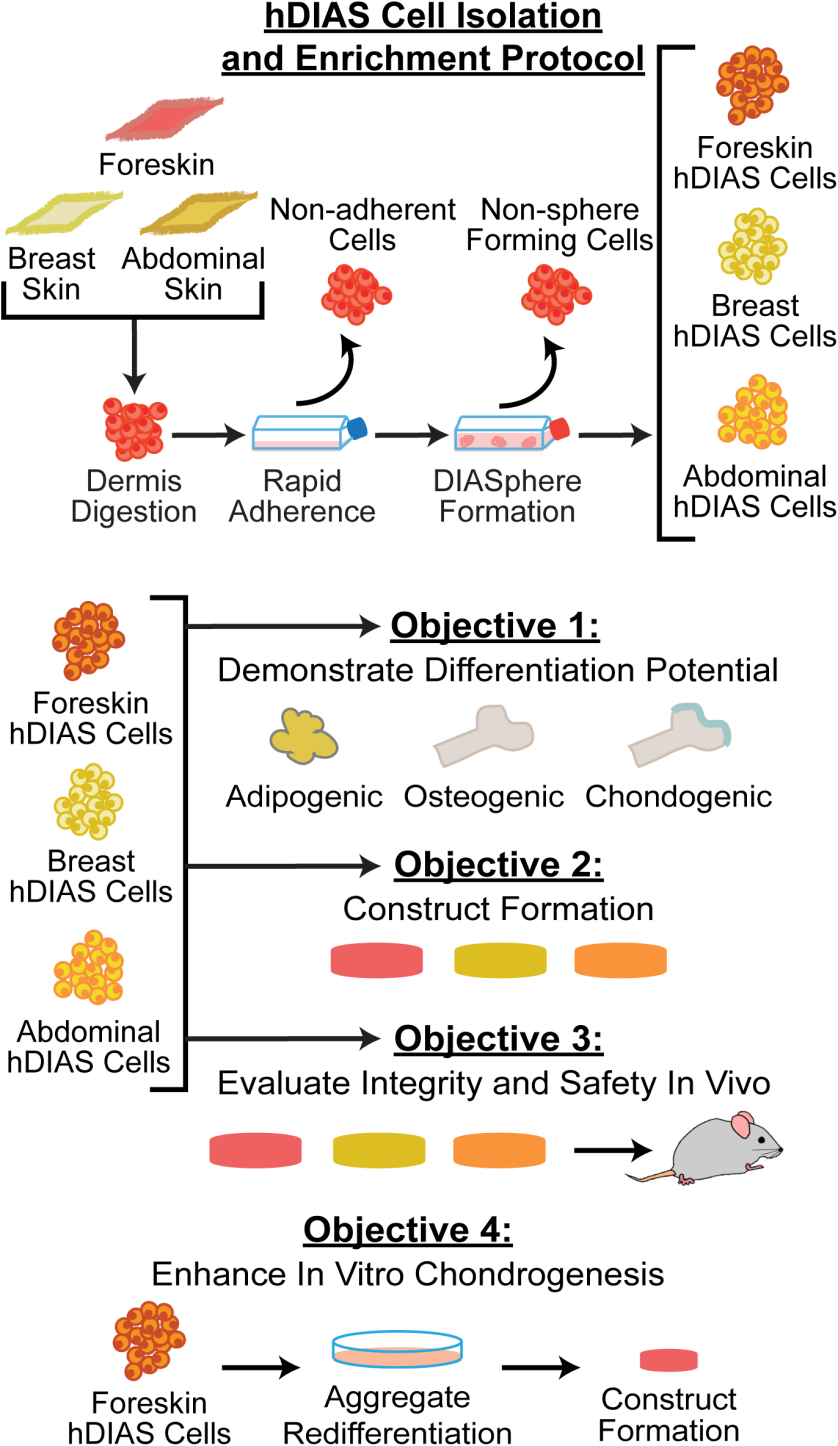
Schematic diagram of the study. Top: isolation and enrichment of hDIAS cells from different anatomical locations; foreskin, breast, and abdomen. Bottom: study objectives; 1) multi-lineage differentiation of hDIAS cells, 2) construct formation using hDIAS cells from different anatomical locations 3) *in vivo* implantation of hDIAS-derived constructs, 4) enhancement of *in vitro* chondrogenesis using aggregate redifferentiation culture.

Dermal cells were thawed and seeded at 2x10^6^ cells per T-225 flask in “expansion medium” consisting of DMEM with high glucose/GlutaMAX™-I, 10% FBS, 1% P/S/F, and 1% non-essential amino acids (NEAA) (Life Technologies). Cells from each anatomical location and donor were processed in parallel in three separate steps (Fig 1). In the first step, human DIAS cells were obtained as previously described [11]. Briefly, cells were lifted using 0.05% trypsin-EDTA (Life Technologies), passaged in T-225 flasks, and allowed to rapidly adhere for 10 min. Non-adherent cells were removed, and the remaining adherent cells were cultured in expansion medium to confluence. In the second step, to enrich this rapidly adhered hDIAS cell population, cells were trypsinized, collected, and seeded using a “sphere colony forming medium” previously described by Biernaskie, et al. [14, 28]. Briefly, hDIAS cells were cultured in medium consisting of DMEM with low glucose/GlutaMAX™-I:F12 with GlutaMAX™-I (3:1) and 1% P/S/F, supplemented with 20 ng/mL epidermal growth factor (EGF) (Peprotech, Rocky Hills, NJ), 40 ng/mL fibroblast growth factor 2 (FGF2) (Peprotech), and 2% B27 (Life Technologies) for 3–4 weeks (DIASphere Formation; Fig 1). The resultant spheres were collected and dissociated in 0.05% trypsin-EDTA. In the third step, the dissociated hDIAS cells underwent a modified chondrogenically tuned expansion step by seeding them at 1x10^6^ cells per T-225 flask in a “chondrogenic medium” (CHG) consisting of DMEM with high glucose/GlutaMAX™-I, 1% P/S/F, 1% NEAA, 1% ITS+ Premix (BD Biosciences, Bedford, MA), 50 μg/mL ascorbate-2-phosphate (Sigma-Aldrich), 40 μg/mL L-proline (Sigma-Aldrich), 100 μg/mL sodium pyruvate (Sigma-Aldrich), and 100 nM dexamethasone (Sigma-Aldrich), supplemented with 10% FBS, 5 ng/mL FGF2 [29]. Cells were further passaged 2–3 times and the resulting cells (foreskin-, breast, and abdominal skin-derived hDIAS cells) were used in subsequent experiments.

### Multi-lineage differentiation

To determine the multi-lineage differentiation potential of cells from each tissue type, hDIAS cells from each donor were grown in established adipogenic, osteogenic, and chondrogenic culture conditions [10]. For adipogenic and osteogenic differentiation, 1.5x10^4^ cells from each donor were plated in each well of a 24-well plate. Adipogenic differentiation medium consisted of DMEM with high glucose/GlutaMAX™-I, 5% FBS, 1% P/S/F, 1% NEAA, 1 μM dexamethasone, 0.5 mM isobutyl methylxanthine (Sigma-Aldrich), and 0.2 mM indomethacin (Sigma-Aldrich). Osteogenic differentiation medium consisted of DMEM with high glucose/GlutaMAX™-I, 10% FBS, 1% P/S/F, 1% NEAA, 100 nM dexamethasone, 10 mM β-glycerophosphate (Sigma-Aldrich), 250 mM ascorbate-2-phosphate, and 50 ng/mL bone morphogenetic protein-2 (BMP-2) (Peprotech). For chondrogenic differentiation, 2.5x10^5^ cells from each donor were added to a round bottom polystyrene 96-well plate (Costar 3799, Corning, NY) 0.2 mL of CHG, supplemented with 50 ng/mL BMP-2, and 10 ng/mL transforming growth factor beta-1 (TGF-β1) (Peprotech), and centrifuged at 500 xg for 5 minutes to form cell pellets [30]. All groups were then maintained at 37°C and 5% CO_2_ for 4 weeks, with media exchanged every other day.

### Self-assembled construct formation

Constructs were formed using the self-assembling process, as described previously [21]. Briefly, 4x10^6^ hDIAS cells derived from each anatomical location were suspended in 100 μL of CHG, supplemented with 10 ng/mL TGFβ1 and 100 ng/mL BMP2. These were seeded in 5 mm-diameter, 2% agarose wells. After 4 hours, 400 μL of CHG supplemented with 10 ng/mL TGFβ1 and 100 ng/mL BMP2 was added to each well. The self-assembled hDIAS constructs were cultured for 4 weeks, and a 1g, porous steel post was set on top of each construct during 2^nd^ and 4^th^ week of culture. Medium was exchanged every day for the first 7 days and every other day throughout the culture period.

### Assessment of safety, stability, and integrity of hDIAS-derived constructs *in vivo*

All animal use was performed with approval of the Institutional Animal Care and Use Committee, University of California, Davis. After 4 weeks of culture in vitro, self-assembled hDIAS cell constructs derived from two different donors of each skin type (i.e., foreskin, breast skin, and abdominal skin) were randomized and implanted subcutaneously (SQ), one on each side of the thorax, in athymic nude mice (Crl:NU(NCr)-Foxn1nu, Charles River Laboratories). Mice were anesthetized with isoflurane (0.5–2%) and oxygen (2L/min) administered by mask. Buprenorphine (0.05 mg/kg SQ) was administered prior to anesthesia to achieve analgesia. Ceftiofur (20mg/kg SQ) was administered at induction and once the mice were anesthetized their cornea were lubricated with ophthalmic ointment. Six mice were used per anatomical location, resulting in a total of 18 mice for the study. Postsurgical care involved daily monitoring of the animals for 10 days by the surgical staff including incision checks for redness, swelling, and oozing, and signs of pain such as reluctance to move, vocalization, anorexia, or excessive tactile attention being paid to an incision site. Post-operative analgesic of buprenorphine was applied at 0.05–0.1 mg/kg SQ twice daily for 72 hours. The mice were humanely sacrificed at 4 weeks with CO_2_ followed by thoracotomy, and the implanted constructs were harvested for assays. Four randomly selected mice were processed for complete histological examination of the organ systems for signs of cardiovascular, central and peripheral nervous, musculoskeletal, respiratory, integument, endocrine, urogenital and gastrointestinal toxicity. All implantation sites were examined histologically for toxicity or neoplasia by a board certified veterinary pathologist (NV). Control groups consisting of self-assembled constructs were maintained *in vitro* in CHG for 4 weeks. Medium was exchanged every other day.

### Histology

All cultured and implanted constructs with associated skin were fixed in 10% neutral buffered formalin, paraffin embedded, sectioned at 5 μm and stained with hematoxylin and eosin (H&E) or Alcian blue, pH = 1.0 and nuclear fast red following standard procedures. The histological specimens were examined and described by a board certified veterinary pathologist (NV). For multi-lineage differentiation, cells in monolayer were fixed and 3D pellets were processed as above. All samples were stained with Alcian blue for chondrogenesis [31], Alizarin red for osteogenesis [32], and Oil red O for adipogenesis [33], as previously described [10].

### Mechanical testing

Creep indentation testing, as previously described [34], was performed at time of implantation and at the end of the 4-week *in vivo* and *in vitro* culture period. Briefly, constructs were first photographed and measured to determine thickness and diameter using ImageJ (NIH, Bethesda, MD) [35]. A porous, flat, stainless steel indenter tip with 0.45 mm radius was applied to each sample under 0.25 g (0.0025 N) load. A semi-analytical, semi-numerical, linear biphasic model and finite element analysis were used to obtain the aggregate modulus, and shear modulus from the experimental data [36].

### *In vitro* aggregate redifferentiation and self-assembly

Human DIAS cells isolated from foreskin were selected based on *in vivo* results. Foreskin-derived hDIAS cells were grown in ARC in which cells were seeded at 7.5x10^6^ cells/mL in petri dishes coated with 1% agarose and cultured in CHG containing 10 ng/mL TGF-β1, 100 ng/mL GDF-5 (Propotech), and 100 ng/mL BMP-2 at 37°C for 7 days on an orbital shaker at 60 rpm [37]. Aggregates were digested with 0.05% trypsin-EDTA for 20 minutes followed by 0.2% collagenase solution containing 3% FBS for 1 hour with agitation every 15 minutes to release cells. The resulting cells were filtered through 70 μm cell strainers and counted. Human DIAS cell constructs using ARC and non-aggregate cultured (control) hDIAS cells were formed via the self-assembling process as described above. For biochemical analysis, lyophilized samples were digested using papain as previously described [38]. Sulfated GAG content was assayed using the Blyscan Glycosaminoglycan Assay kit (Biocolor, Westbury, NY). Total collagen content was quantified using a hydroxyproline assay (Biocolor) [39]. DNA content was determined using the PicoGreen® dsDNA Assay Kit (Life Technologies) [40]. Mechanical testing was performed as described above.

### Statistical analysis

All mechanical evaluations in this study were performed using n = 4–6 constructs per group. All biochemical evaluations were performed using n = 6 per group. All numerical data are represented as mean ± standard deviation. For comparisons of three or more groups, statistical analysis was performed using one-way ANOVA with Tukey’s *post-hoc* analysis, when warranted, using JMP v12 (SAS, Cary, NC), with p<0.05 indicating statistical significance. Significant differences are indicated by bars not sharing the same letter. For comparisons between two groups, a Student’s t-test was used to determine significance. Significance (p<0.05) is indicated by an asterisk.

## Results

### Multi-lineage differentiation of hDIAS cells isolated from various anatomical locations

**Fig 2:**
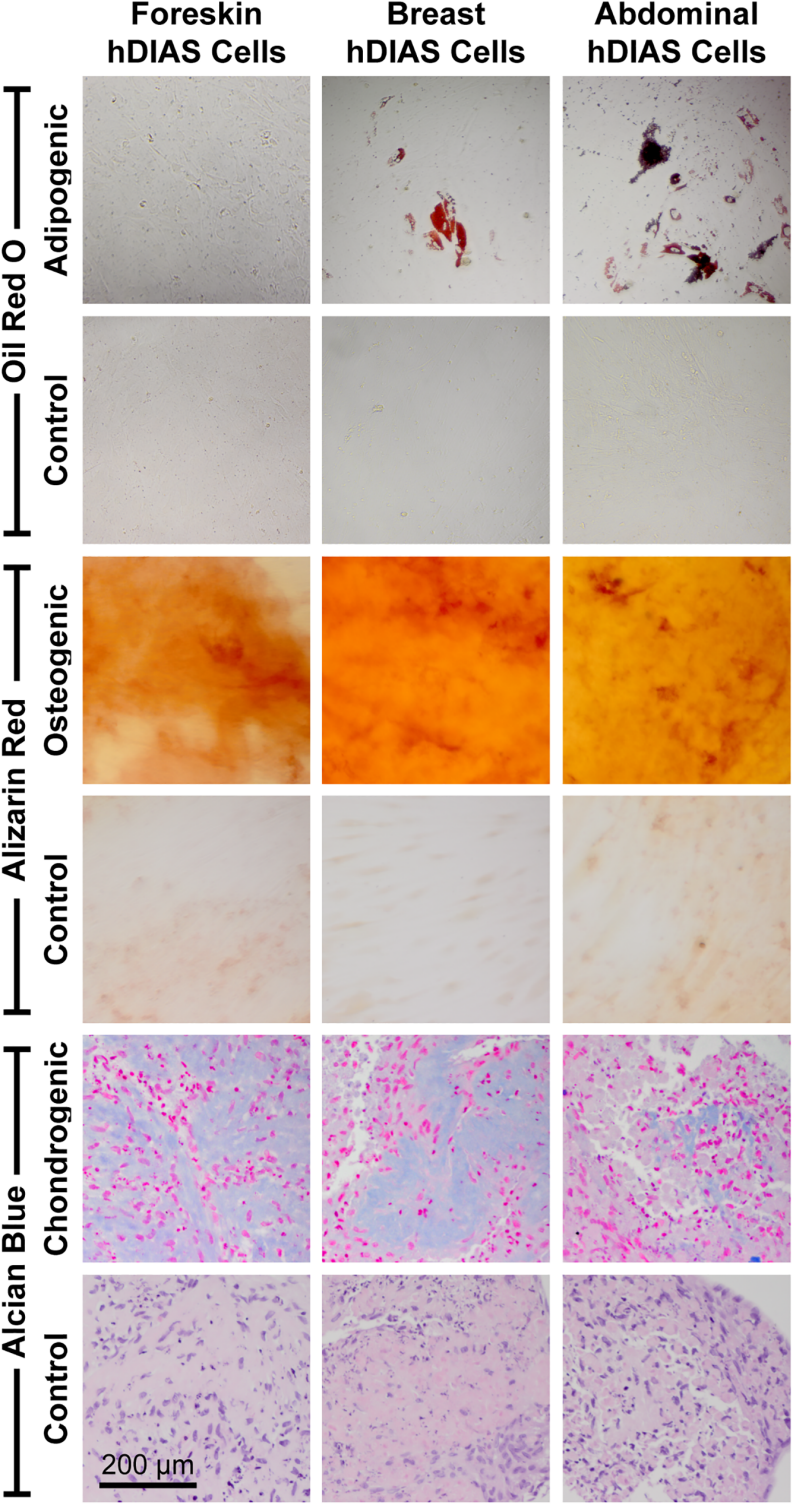
Multi-lineage differentiation of hDIAS cells derived from foreskin, breast, and abdomen. Human DIAS cells from each anatomical location were exposed to adipogenic, osteogenic, and chondrogenic differentiation media for 4 weeks. Top: Oil red O staining for adipogenesis. Middle: Alizarin red staining for osteogenesis. Bottom: Alcian blue staining for chondrogenesis.

Human DIAS cells displayed the potential to differentiate along multiple lineages regardless of the anatomical location of the source tissue (Fig 2). Foreskin-, breast, and abdominal skin-derived hDIAS cells all stained positive for Alizarin red after 4 weeks of monolayer culture in osteogenic medium. Human DIAS cell pellets from all three skin sources also stained positive for Alcian blue after being cultured in chondrogenic medium. Interestingly, only breast and abdominal skin-derived hDIAS cells stained positive for Oil red O when cultured in adipogenic medium. Foreskin-derived hDIAS cells did not stain with Oil red O. Foreskin-derived hDIAS cells from two additional donors were processed also without evidence of adipogenic differentiation (S1 Fig). Ranges for cell yields were 2.05–2.40, 2.65–3.45, and 1.45–7.83 million cells per gram of foreskin, breast skin, and abdominal skin, respectively.

### *In vivo* assessment of safety and toxicity

After 4 weeks of culture *in vitro* (referred to as t = 0 w), human foreskin, breast, and abdominal skin-derived constructs were implanted subcutaneously in mice for an additional 4 weeks (referred to as t = 4 w) before removal and assessment for phenotypic stability, mechanical integrity, and safety. The incision sites healed without complications and surgical staples were removed 1 week after surgery. No functional or behavioral abnormalities were detected during the post-operative period. Postmortem, animals received detailed gross examination, and no abnormalities were detected in any of the mice. No abnormalities were detected in any of the organ systems examined. Histological examination of all implantation sites revealed no evidence of toxicity or neoplasia.

### Gross morphology and mechanical properties of constructs pre- and post-implantation

**Fig 3:**
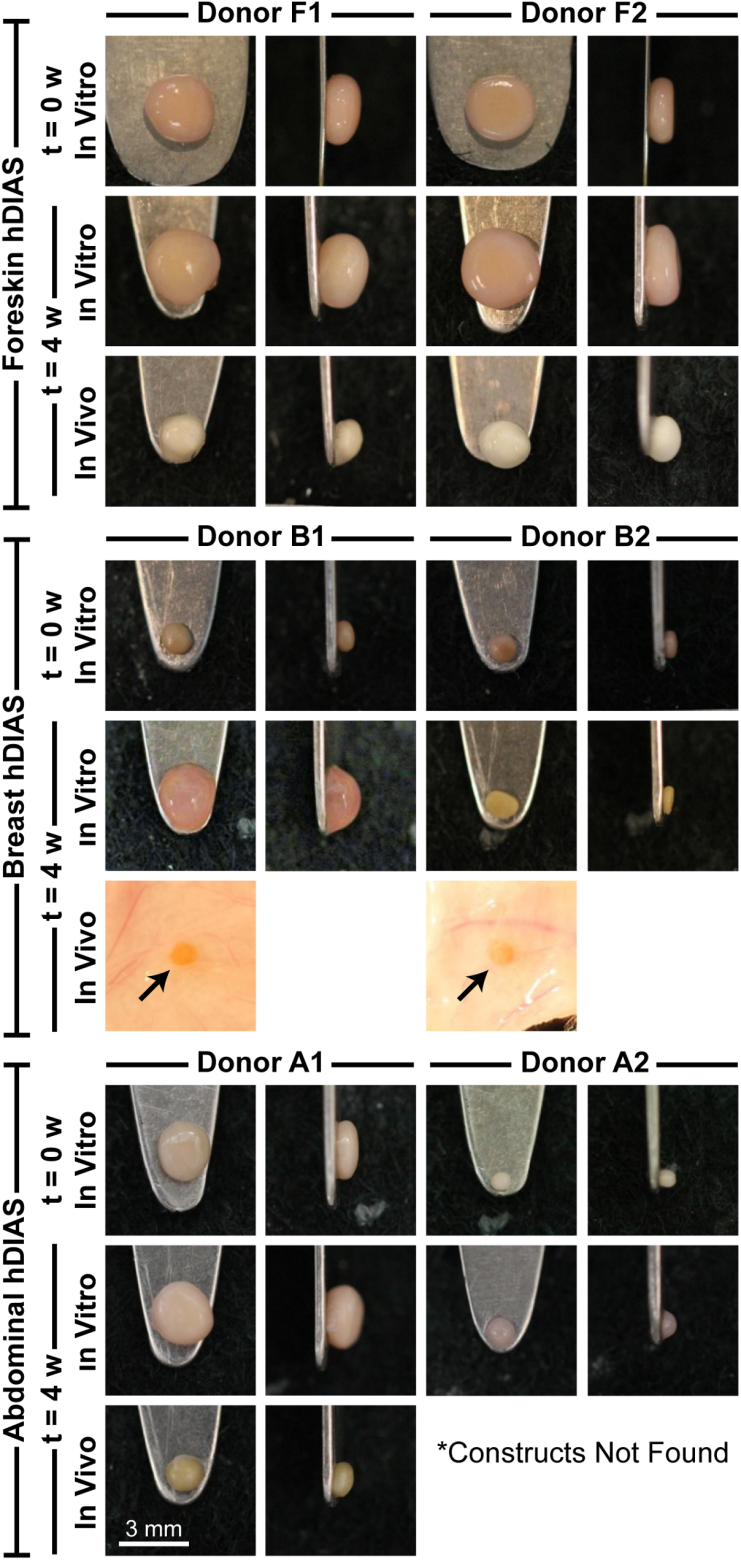
Gross morphology of hDIAS-derived constructs. Shown is gross morphology of top and side views of constructs derived from two different donors for each anatomical location (foreskin, breast, and abdomen), at the time of implantation (referred to as t = 0 w *in vitro*), after 4 weeks *in vitro* culture (referred to as t = 4 w *in vitro*), after 4 weeks *in vivo* culture (referred to as t = 4 w *in vivo*). Breast skin-derived constructs at t = 4 w *in vivo* are shown with the surrounding host skin due to size of the constructs. Abdominal skin-derived constructs from donor A2 were not found at t = 4 w *in vivo*.

Self-assembled hDIAS cell constructs derived from each human anatomical location exhibited distinctly different gross morphologies (Fig 3). Foreskin-derived constructs were of uniform size and shape, regardless of donor. When seeding breast skin-derived hDIAS cells to form self-assembled constructs, not all seeded cells were incorporated into the final construct. A loose, disperse plume of cells formed adjacent to the construct; these loose cells were removed upon medium exchange, resulting in breast skin-derived constructs that were significantly (p<0.001) smaller in size than those formed using other hDIAS cells. Abdominal skin-derived hDIAS cells from donor A1 self-assembled into a construct with similar gross morphology as constructs formed using foreskin-derived hDIAS cells. Similar to breast skin-derived hDIAS cells, not all abdominal skin-derived hDIAS cells from donor A2 were incorporated into the final construct.

After *in vivo* culture, the average diameter of foreskin-derived constructs was significantly reduced from 3.1±0.1 mm to 2.2±0.1 mm (t = 0 w and t = 4 w *in vivo*, respectively; p<0.001). The decrease in size of breast skin-derived constructs from 1.3±0.2 mm to less than 1 mm made retrieval without damage impossible; these were embedded and sectioned for histology *in situ* without evaluation of their mechanical properties. The size of abdominal skin-derived constructs from donor A1 decreased from 2.5±1.0 mm to 1.7±0.5 mm in diameter. Constructs formed using abdominal skin-derived hDIAS cells from donor A2 were not found at the implant site. *In vitro* control constructs formed using foreskin-derived hDIAS cells maintained a diameter of 3.4±0.2 mm (average of both donors). *In vitro* control constructs formed using breast skin-derived hDIAS cells from donor B1 increased in size to 2.3±0.7 mm, while no distinct difference was observed in size or morphology for constructs from donor B2. After *in vitro* control culture, the average diameter of abdominal skin-derived constructs was comparable from 2.5±1.0 mm to 2.6±0.9 mm (t = 0 w and t = 4 w *in vitro*, respectively). It should be noted that, unlike constructs derived from foreskin-derived hDIAS cells, breast and abdominal skin-derived constructs displayed large variability in size and morphology across donors (refer to S2 Table for the average size of hDIAS-derived constructs).

### Histological evaluation of constructs pre- and post-implantation

**Fig 4:**
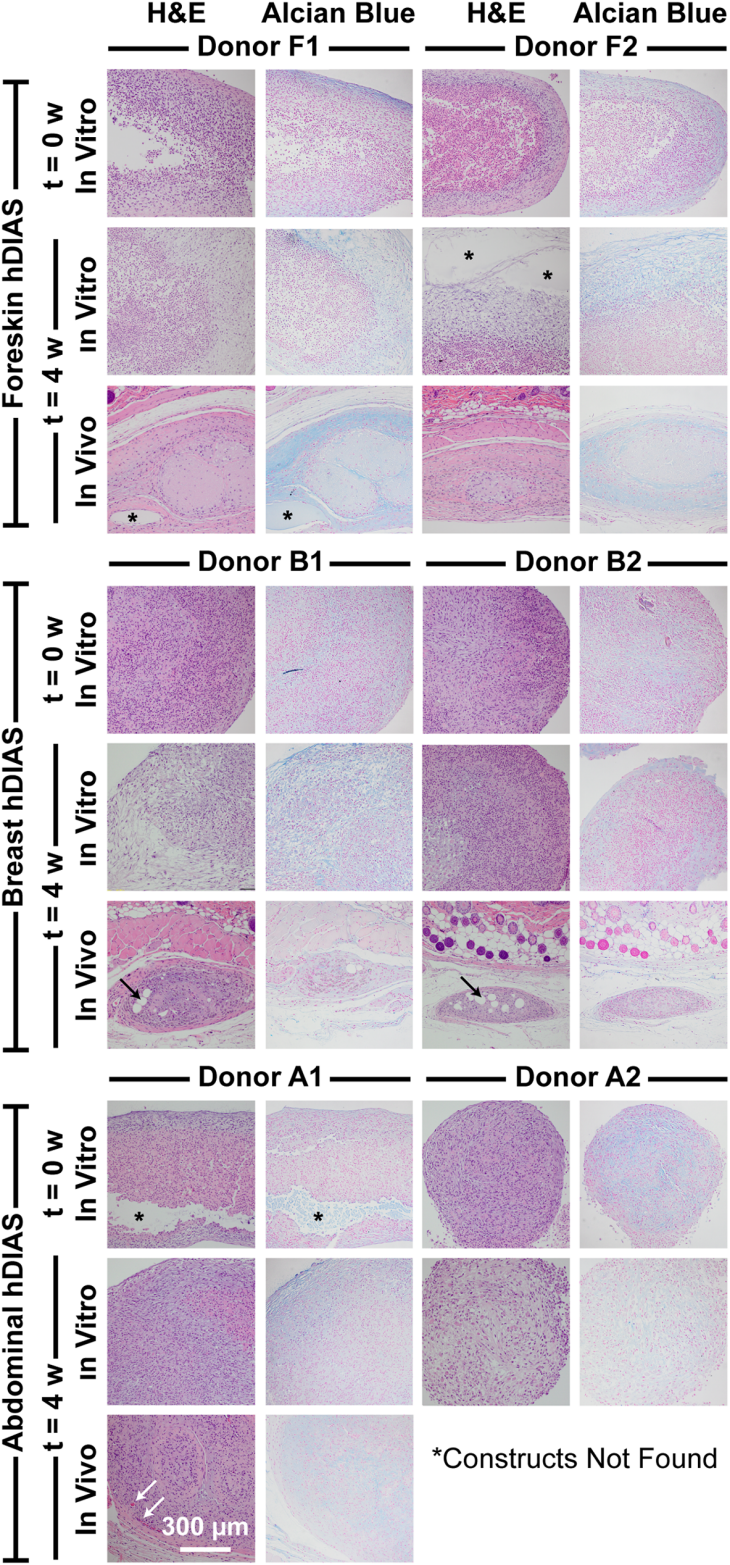
Histological evaluation of hDIAS-derived constructs. Shown are H&E and Alcian blue stains of hDIAS constructs derived from two different donors for each skin type (foreskin, breast, and abdomen), at the time of implantation (t = 0 w *in vitro*), after 4 weeks *in vitro* culture (t = 4w *in vitro*), after 4 weeks *in vivo* culture (t = 4 w *in vivo*). No histological evaluation of abdominal skin-derived constructs from donor A2 were available because constructs were not found at t = 4 w *in vivo*. Asterisks indicate acellular areas within constructs. The arrows point at adipose cells within breast skin implants and blood vessel capillaries in abdominal skin implants derived from donor A1 *in vivo*.

At t = 0 w, construct histological characteristics appeared similar regardless of anatomical location or donor; each contained two distinct cellular populations as evidenced by their morphologies based on H&E staining (Fig 4). Externally, cells formed a capsule-like structure containing loose extracellular matrix (ECM), surrounding a second population of cells with eccentric nuclei that formed non-coalesced aggregates without ECM. Alcian blue staining was present in the ECM deposited around cells in the capsule-like structure.

Upon an additional 4 weeks of culture *in vitro*, the capsule-like component of the constructs progressively thickened due to expansion of the ECM component, as evident from H&E evaluation. Occasional pockets containing homogeneous or granular acellular substance were observed in both foreskin- and abdominal skin-derived constructs (Fig 4; asterisks). As observed in the constructs at t = 0 w, Alcian blue staining highlighted the ECM component deposited in the outer area of constructs, regardless of skin anatomical location. No significant histological differences were detected among the constructs derived from examined donors.

After 4 weeks of subcutaneous implantation, constructs derived from all skin anatomical locations resulted in significant size reduction from t = 0 w. The capsule-like structures of all constructs remained viable and exhibited progressive ECM maturation; mature collagen fibers were observed at 4 weeks after implantation (Fig 4). The non-coalesced aggregates seen at t = 0 w became acellular cores after *in vivo* culture. Macrophages were frequently found infiltrating the implants. The macrophage cytoplasm was often expanded and discolored gray, suggestive of phagocytic activity. Adipose cells were occasionally present in breast skin-derived constructs (Fig 4; black arrows), while rare capillaries were observed within abdominal skin-derived constructs (Fig 4; white arrows). Foreskin-derived constructs exhibited relatively stronger Alcian blue staining after *in vivo* culture. There was no evidence of toxicity to the surrounding tissues and no capsule formation around the implants was discernible for hDIAS cells regardless of anatomical location or donor. No abnormalities were seen in the host’s organs during necropsy.

### Mechanical evaluation of constructs pre- and post-implantation

**Fig 5:**
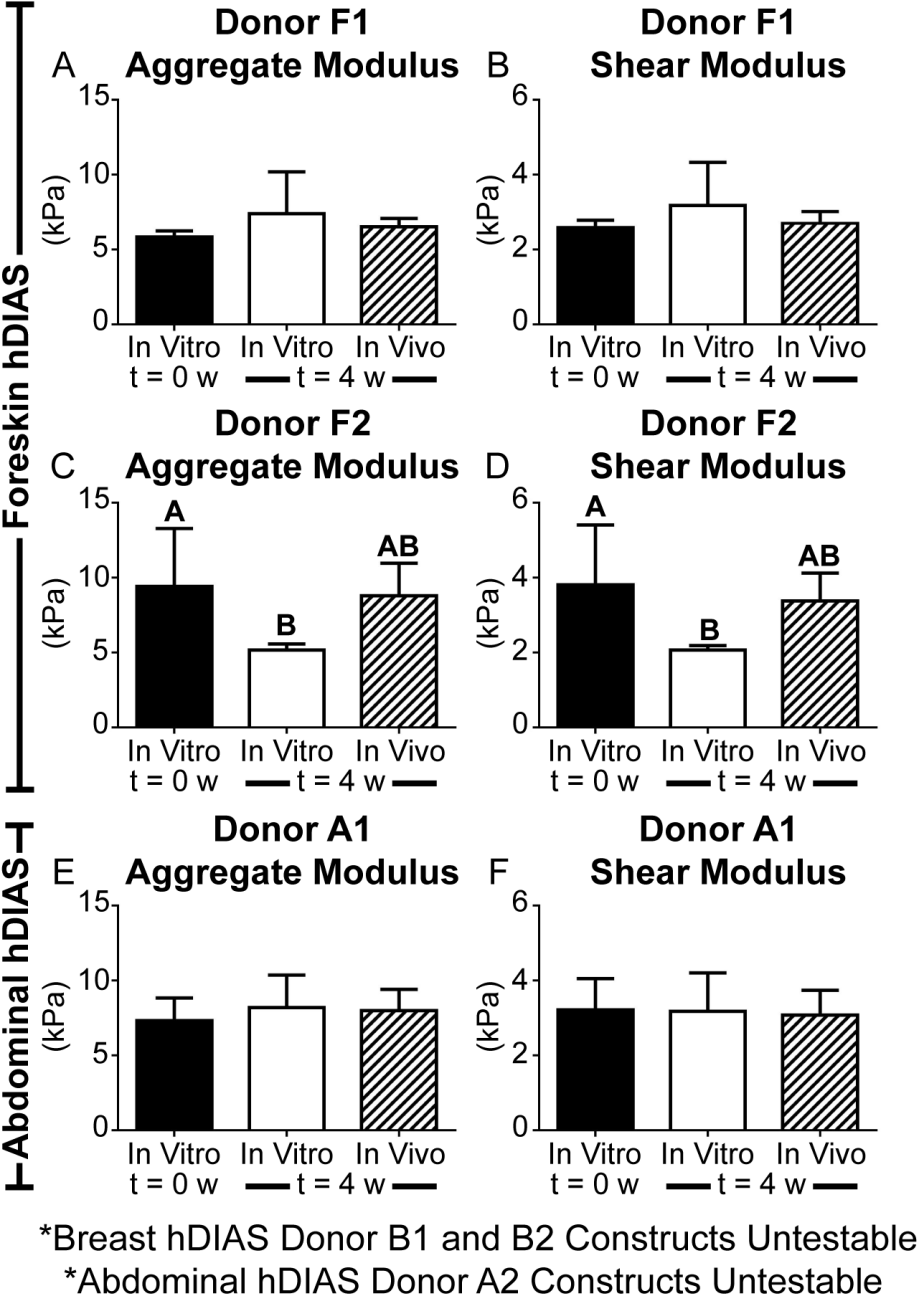
Mechanical properties of hDIAS-derived constructs. Shown are aggregate modulus and shear modulus of hDIAS constructs derived from two different donors of foreskin and donor A1 of abdominal skin at the time of implantation (t = 0w *in vitro*), after 4 weeks *in vitro* culture (t = 4w *in vitro*), after 4 weeks *in vivo* culture (t = 4w *in vivo*). Breast skin and donor A2’s abdominal skin-derived constructs were mechanically untestable due to construct size.

While all constructs exhibited sufficient handling capability to be implanted at t = 0w, abdominal skin-derived constructs from donor A2 and breast skin-derived constructs were uniformly too small to be mechanically tested. Biomechanical data for all testable constructs at t = 0 w, and for constructs cultured an additional 4 weeks *in vivo* or *in vitro* are presented in Fig 5. Although implantation of the constructs *in vivo* resulted in reduction in size from t = 0 w, the mechanical integrity of all implanted constructs was not compromised after 4 weeks *in vivo*. The aggregate modulus and shear modulus of foreskin-derived constructs from donor F1 did not change significantly after an additional 4 weeks of *in vivo* implantation or *in vitro* culture. The mechanical properties for foreskin-derived constructs from donor F2 were retained with *in vivo* culture while *in vitro* culture resulted in a significant decrease in aggregate modulus (p<0.03; Fig 5) and shear modulus (p<0.03; Fig 5). Following *in vivo* and *in vitro* culture, the mechanical properties of abdominal skin-derived constructs from donor A1 were comparable to properties at t = 0 w. Constructs derived from foreskin, and abdomen exhibited similar mechanical properties.

### *In vitro* chondrogenic aggregate redifferentiation of foreskin-derived hDIAS cells

**Fig 6:**
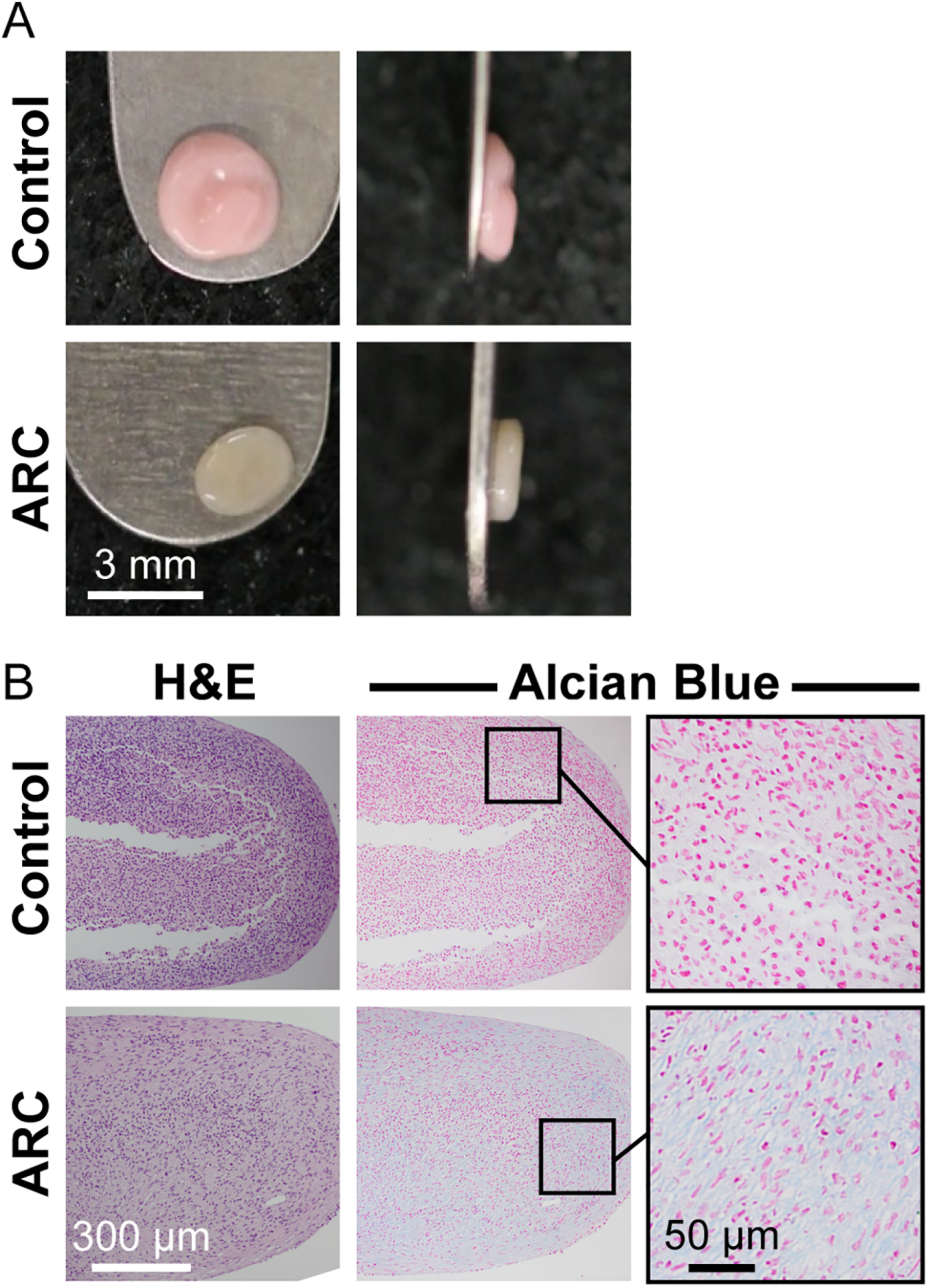
*In vitro* chondrogenesis of foreskin-derived hDIAS cells in aggregate culture. A: gross morphology of foreskin-derived constructs from untreated hDIAS cells (referred to as control) and hDIAS cells that underwent aggregate redifferentiation culture (referred to as ARC). B: H&E and Alcian blue staining of foreskin-derived constructs from untreated hDIAS cells (control) and hDIAS cells that underwent ARC.

Human DIAS cells isolated from foreskin were chosen based on size, donor consistency, and handling properties. Cells were seeded into ARC at 110x10^6^ cells. Digestion of the aggregates after 7 days resulted in a 77.2% cell yield. Cells which underwent ARC formed self-assembled hDIAS constructs of significantly smaller diameter than control constructs composed of hDIAS cells which did not undergo ARC (2.3±0.4 mm and 3.0±0.4 mm, respectively; *p*<0.003) (Fig 6). During self-assembly, not all foreskin-derived hDIAS cells that underwent ARC self-assembled into constructs; a loose cloud of cells was seen around the engineered construct. This is in contrast to untreated foreskin-derived hDIAS cells, which all coalesced into a single construct. Cells that were not incorporated into the construct were removed during medium exchange.

**Fig 7:**
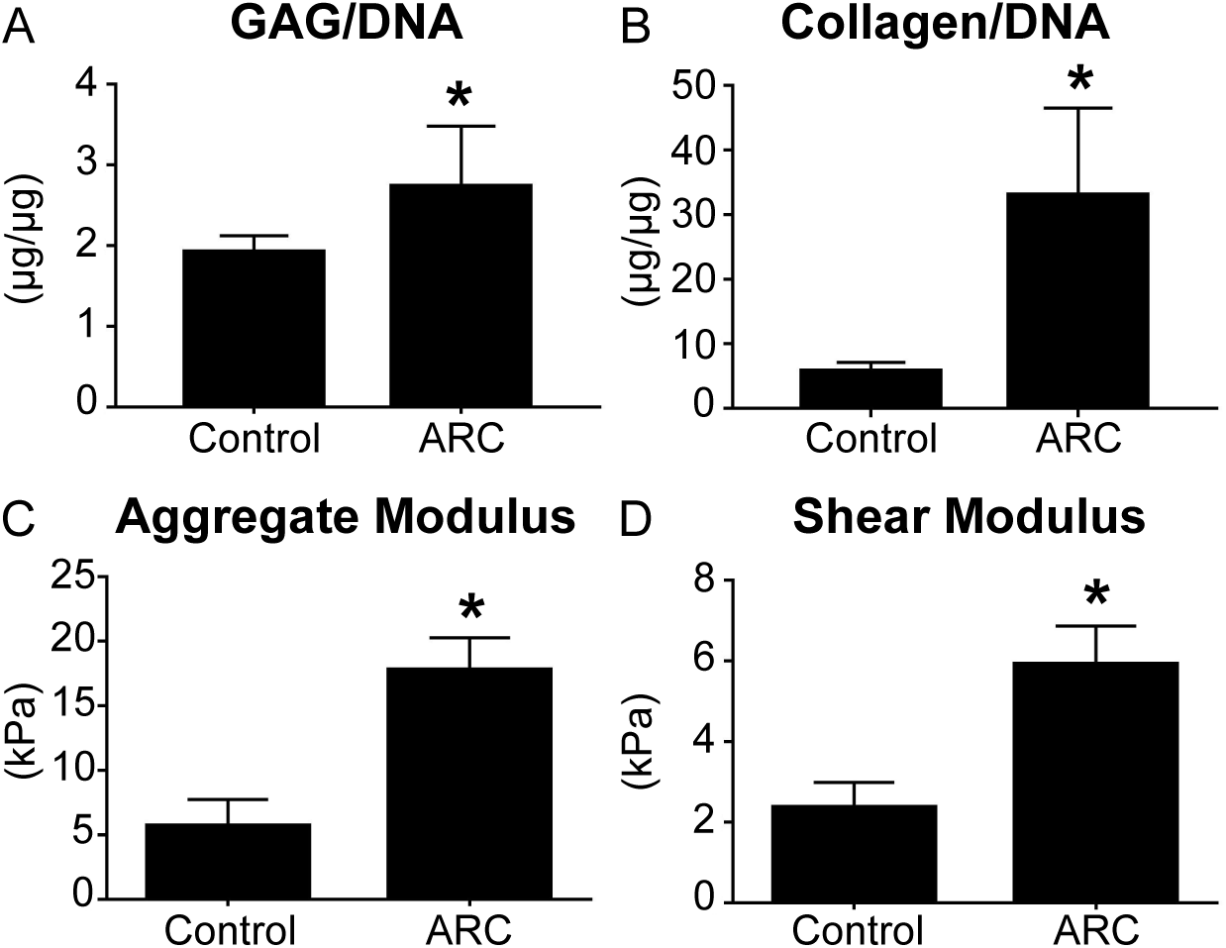
The effect of aggregate redifferentiation culture *in vitro* on construct biochemical and mechanical properties. Biochemical and mechanical properties were compared between foreskin-derived constructs from untreated hDIAS cells (control) and hDIAS cells that underwent ARC. A: GAG content normalized to DNA. B: collagen content normalized to DNA. C: aggregate modulus. D: shear modulus.

ARC enhanced foreskin-derived hDIAS cell construct functional properties. Control constructs had irregular surfaces and were less robust than the group that underwent ARC, which produced flat discs. ARC induced more intense Alcian blue staining in the hDIAS cell constructs over control (Fig 6). Wet and dry weights of the control constructs were significantly greater than the constructs that underwent ARC (wet weight: 4.3±0.4 mg and 2.5±0.2 mg, respectively; p<0.001; dry weight: 1.0±0.1 mg and 0.4±0.1 mg, respectively; p<0.001). Biochemically (Fig 7A and 7B), ARC significantly increased GAG per DNA (from 1.9±0.2 μg/μg in controls to 2.7±0.7 μg/μg with ARC; p<0.05) and collagen per DNA (from 5.8±1.3 μg/μg to 33.1±13.4 μg/μg; p<0.001). Mechanically (Fig 7C and 7D), ARC significantly increased the compressive aggregate and shear modulus values of the constructs over those of untreated controls (17.8±2.5 kPa vs. 5.7±2.1 kPa; p<0.001 and 5.9±1.0 kPa vs. 2.4±0.6 kPa; p<0.001, respectively).

## Discussion

The advantages conferred by skin-derived stem cells over other stem cells include accessibility, abundance, and the potential for autologous therapies. Skin-derived stem cells, isolated from animal skin and from human foreskin, have been used in attempts to engineer tissues other than skin, although questions remain with regard to translatability. Using tissue engineered articular cartilage as a model system, this study produced several key findings: 1) human skin-derived stem cells from different anatomical locations possess the potential to differentiate toward multiple lineages, 2) anatomical location alters engineered construct properties, 3) skin-derived stem cells from different anatomical locations are all safe and stable, and maintain mechanical integrity *in vivo*, and 4) with respect to translation of established chondroinductive protocols to human cells, foreskin-derived hDIAS cells respond to ARC efficaciously.

The multi-lineage differentiation potential of human skin-derived stem cells varied with anatomical location. Previous studies on cultured human foreskin and breast skin primary fibroblasts showed that human skin fibroblasts do not undergo any multi-lineage differentiation after 28 days in induction media [10]. By utilizing a combination of rapid adherence [41], and DIASphere formation [14], this study found that the resulting hDIAS cell populations isolated from foreskin, breast, and abdominal skins all exhibited multi-lineage differentiation potential. All populations exhibited osteogenic and chondrogenic differentiation under appropriate culture conditions (Fig 2). The adipogenic potential of hDIAS cells was dependent on the anatomical location of the source tissue. Human DIAS cells isolated from breast and abdominal skins both exhibited adipogenic differentiation while foreskin derived hDIAS cells did not stain for Oil Red O after 4 weeks of culture in adipogenic medium, regardless of donor. Similarly, it has been previously shown that human umbilical cord blood-derived mesenchymal stem cells differentiate easily into an osteogenic lineage but not an adipogenic lineage [42]. Previous studies using human foreskin-derived precursors that were cultured in sphere colony forming medium without rapid adherence or chondrogenically tuned expansion steps were capable of forming adipogenic lipid droplets in the presence of retinoic acid [14]. The lack of adipogenic potential of foreskin-derived hDIAS cells in this study may be a result of the additional cell processing steps or the medium composition being insufficient for this particular cell type. Overall, the finding that hDIAS cells derived from abdominal and breast skins do not vary in their multi-linage potential bodes well for the potential of autologous therapies based on these cells.

While hDIAS cells from all anatomical locations and donors were capable of expansion, sphere formation, and self-assembly into constructs, skin location- and donor-associated variations resulted in a range of construct sizes and morphologies. It has previously been shown that the frequency of stem cells in the stromal vascular fraction of adipose tissues derived from abdomen was significantly higher than those from the hip/thigh region, although their potential for chondrogenic and osteogenic differentiation were similar [43]. Furthermore, human synovium stem cells derived from four different anatomical sites contained different numbers of CD31- positive cells and α-smooth muscle actin-positive cells which were correlated with their colony-forming ability [44]. With respect to donor variation, it has been shown that human skin-derived precursors (SKP) cells isolated from multiple anatomical locations exhibited an age-dependent decrease in abundance and differentiation potential [45]. These previous studies suggest that hDIAS cells derived from different anatomical locations may contain different quantities of stem cells which could have resulted in variations in construct formation. Furthermore, the difference noted in formation of abdominal skin-derived constructs suggests that there may be donor-to-donor variations. Thus, it is plausible that the number of stem cells present in each anatomical location and donor could vary, and that these variations could result in constructs of different size and shape. To fully determine the role of donor-based variations, in future studies it may be necessary to characterize stem cell populations in hDIAS cells derived from different anatomical locations using multiple donors. Furthermore, it may be necessary to investigate the correlations between hDIAS cell populations identified by cell surface markers with chondrogenic potential and eventual tissue formation capacity.

There are biological properties of human skin that differ based on anatomical location, age, sex, and race. For example, groups have shown differences in the size and distribution of hair follicles [46], which are an important reservoir for stem cells in the human body [47]. Differences in dermal thickness based on age, sex, and health have also been reported [48]. While the mechanical or matrix properties of the skin were not tested prior to cell isolation, qualitative differences of the tissue were observed during handling and may have affected the function of isolated cells. In terms of associated fat and dermal thickness, foreskin was noticeably thinner and possessed less associated fat than abdominal or breast skin.

Evaluating biocompatibility of stem cell-based constructs, using small animal models, informs future studies, leading to eventual clinical application. Human constructs derived from foreskin, breast, and abdominal skins elicited no adverse host responses, were stable, and maintained integrity *in vivo*. Previous research has also shown that human adult skin-derived dermal fibroblasts possess an immunosuppressive capacity equivalent to bone marrow mesenchymal stem cells [49], which supports our results for the safety assessment of hDIAS cells. Histological evaluation after implantation showed macrophage infiltration and formation of mature collagen fibers indicating the possibility of ECM remodeling within the constructs. The inability to recover and mechanically test any breast skin-derived constructs or donor A2’s abdominal skin-derived constructs following *in vivo* implantation, is most likely due to macrophage-mediated absorption and remodeling. At the implant site, adipose cells and small caliber blood vessels were observed within the implants. Similar results were observed in skin-derived precursors derived from rodent foreskin which differentiated into adipocytes in response to the local microenvironment *in vivo* [50]. Considering that a well-known property of mesenchymal stem cells is that they are strongly affected by the surrounding microenvironment [51], it would be plausible to assume that the implanted hDIAS cells may exhibit a more chondrogenic phenotype if placed in a more clinically relevant osteochondral defect model, as opposed to subcutaneous implantation.

ARC has been used to promote the chondrogenic redifferentiation of expanded chondrocytes and has shown promise for the chondrogenic stimulation of human stem cells. Used post-expansion with TGF-β1 stimulation, ARC enabled passage 7 leporine articular chondrocytes to form neocartilage with biochemical and mechanical properties on par with self-assembled neocartilage composed of passage 2 cells [52]. Furthermore, ARC has been used to promote the chondrogenesis of human mesenchymal stem cells, inducing the upregulation of chondrogenic markers COL2A1, SOX9, and ACAN [37]. Similarly, this study showed that the addition of an ARC step immediately following DIASphere formation could significantly improved both the biochemical and mechanical properties of self-assembled hDIAS constructs. Grossly, despite their smaller size, ARC constructs were more robust upon handling, stained more intensely with Alcian Blue staining (Fig 6), and had significantly increased GAG, and total collagen per DNA (Fig 7). These changes in biochemical content also positively affected the mechanical properties of the constructs. Aggregate modulus increased 2-fold and shear modulus 1.5-fold in constructs composed of cells that underwent ARC. The improvement of chondrogenic functional properties supports the use of ARC as an effective technique to promote the chondrogenic differentiation of hDIAS cells. It is worth noting that the constructs formed after ARC were also smaller than untreated controls. This may be explained by the observation that, during construct self-assembly, cells that underwent ARC separated into two populations, one that was incorporated into the construct, and another of loose cells that were removed upon medium exchange. Following ARC in this study, a 22.8% decrease in hDIAS cell number was also observed. Given that cell death has been reported previously in MSCs undergoing aggregate suspension culture despite the upregulation in COL2 and ACAN [53], the results seen in this study were not unexpected. Indeed, it may be that the enhancement of chondrogenic functional properties seen here was due to an elimination of cells that are unfit to participate in the chondrogenic self-assembling process.

## Conclusion

The objective of this study was to assess the utility of skin-derived stem cells as a potential cell source using tissue engineered articular cartilage as a model system. We demonstrated that hDIAS cells derived from various anatomical locations showed multi-lineage differentiation potential, though hDIAS cells derived from foreskin had limited ability for adipogenic differentiation. Interestingly, distinctively different characteristics in construct formation were observed as a function of anatomical location. Constructs demonstrated stability, integrity, and no adverse reactions in host animals. This work demonstrated for the first time, the use of human skin-derived stem cells from different anatomical locations in a tissue engineering application, and the stability, integrity, and safety of the resulting tissue *in vivo*. Furthermore, applying ARC improved the chondrogenic potential of skin-derived stem cells, signifying that this and other chondroinductive protocols have translation potential to hDIAS cells. Taken together, these results suggest that human skin-derived stem cells, isolated from a variety of easily accessible locations, can serve as potential cell sources for multiple tissue engineering applications.

## Supporting information

S1 Fig Adipogenic differentiation of foreskin-derived hDIAS cells. Foreskin-derived hDIAS cells from three different donors were exposed to either control medium, consisting of DMEM with high glucose/GlutaMAX™-I, 10% FBS, 1% NEAA, and 1% P/S/F, or adipogenic differentiation medium, consisting of control medium with added 1 μM dexamethasone, 0.5 mM isobutyl methylxanthine, 0.2 mM indomethacin, and reduction to 5% FBS. After 4 weeks of culture, cells were stained with Oil red O for adipogenesis.
(TIF)

S1 Table Characteristics of human skin employed in the study, in terms of source, age, sex, and ethnicity. (PDF)

S2 Table Size of hDIAS-derived constructs, in terms of skin type, culture conditions, and time in culture. (PDF)
