# Supplementary figures and images for: Tissue engineering potential of human dermis-isolated adult stem cells from multiple anatomical locations
Source: PLoS One. 2017 Aug 2;12(8):e0182531. doi: 10.1371/journal.pone.0182531 (PMC5540597; doi:10.1371/journal.pone.0182531)

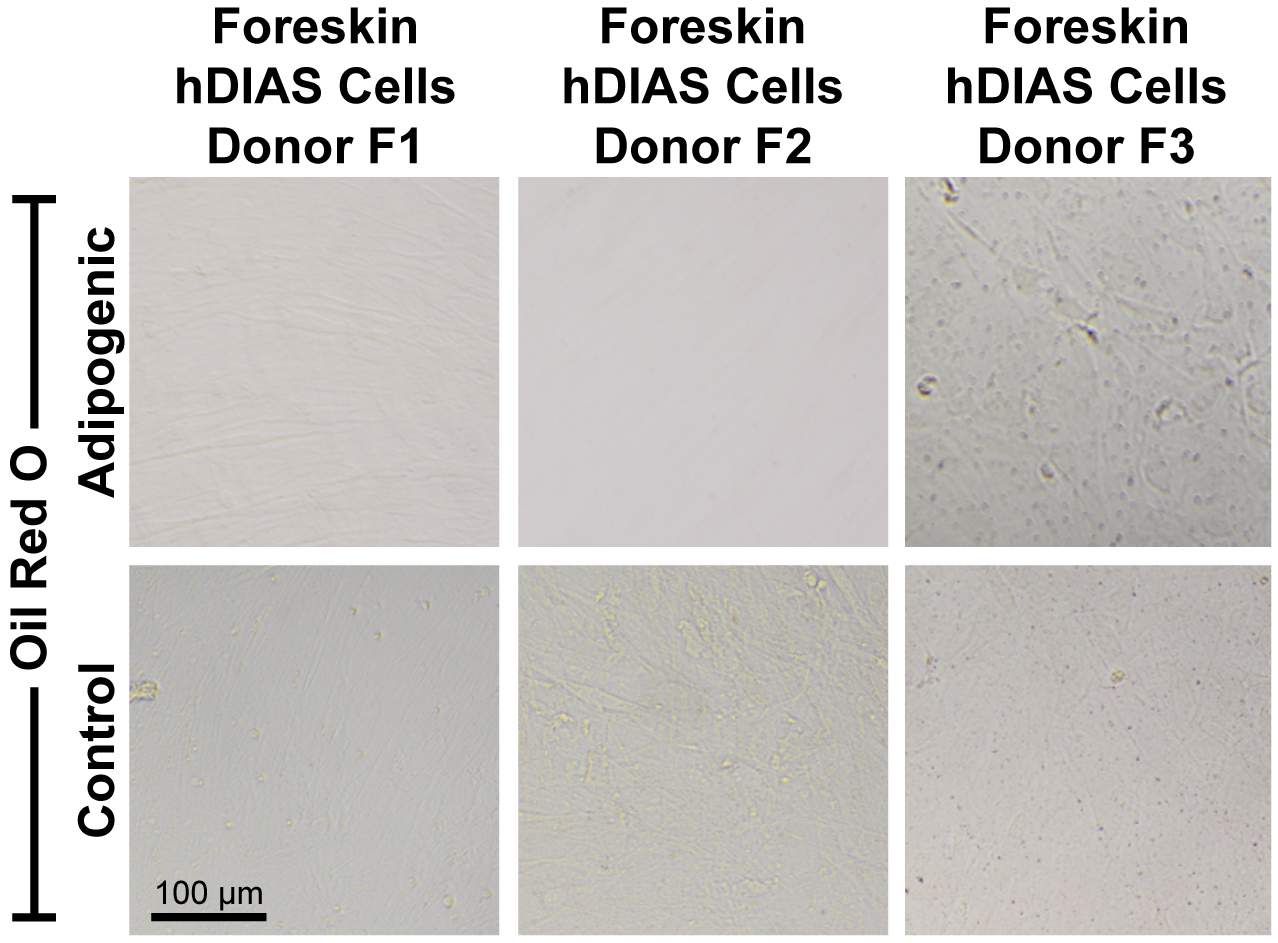

Supplement: S1 Fig — Foreskin-derived hDIAS cells from three different donors were exposed to either control medium, consisting of DMEM with high glucose/GlutaMAX™-I, 10% FBS, 1% NEAA, and 1% P/S/F, or adipogenic differentiation medium, consisting of control medium with added 1 μM dexamethasone, 0.5 mM isobutyl methylxanthine, 0.2 mM indomethacin, and reduction to 5% FBS. After 4 weeks of culture, cells were stained with Oil red O for adipogenesis. (TIF) [file pone.0182531.s001.tif]
