# Supplementary material for: Tissue engineering potential of human dermis-isolated adult stem cells from multiple anatomical locations
Source: PLoS One. 2017 Aug 2;12(8):e0182531. doi: 10.1371/journal.pone.0182531 (PMC5540597; doi:10.1371/journal.pone.0182531)
Supplement: S1 Table — (PDF) [file pone.0182531.s002.pdf]

**Tissue Source**	**Donor ID**	**Age (Years)**	**Sex**	**Ethnicity**
In Vivo Evaluation of Integrity and Safety				
Foreskin	F1	0	Male	Caucasian
	F2	0	Male	Caucasian
Breast Skin	B1	32	Female	Caucasian
	B2	22	Female	African American
Abdominal Skin	A1	32	Female	Caucasian
	A2	54	Female	African American
In Vitro Enhancement of Chondrogenesis				
Foreskin	F3	0	Male	Caucasian
