# Supplementary material for: Tissue engineering potential of human dermis-isolated adult stem cells from multiple anatomical locations
Source: PLoS One. 2017 Aug 2;12(8):e0182531. doi: 10.1371/journal.pone.0182531 (PMC5540597; doi:10.1371/journal.pone.0182531)
Supplement: S2 Table — (PDF) [file pone.0182531.s003.pdf]

Skin Type	Culture	Time point	Diameter (mm)	Thickness (mm)
Abdominal Skin	In-vitro	0 w	2.47 ± 1.04	0.99 ± 0.20
Abdominal Skin	In-vitro	4 w	2.60 ± 0.87	1.24 ± 0.43
Breast Skin	In-vitro	0 w	1.25 ± 0.15	0.61 ± 0.12
Breast Skin	In-vitro	4 w	2.28 ± 0.71	0.79 ± 0.37
Foreskin	In-vitro	0 w	3.12 ± 0.09	1.34 ± 0.29
Foreskin	In-vitro	4 w	3.40 ± 0.15	1.92 ± 0.29
Abdominal Skin	In-vivo	4 w	1.72 ± 0.24	0.94 ± 0.20
Foreskin	In-vivo	4 w	2.20 ± 0.50	1.04 ± 0.19
